# A novel model of urosepsis in rats developed by injection of Escherichia coli into the renal pelvis

**DOI:** 10.3389/fimmu.2022.1074488

**Published:** 2023-01-05

**Authors:** Yuanfei Cao, Can Bai, Penghui Si, Xin Yan, Peng Zhang, Zuhaer Yisha, Peixiang Lu, Kuerban Tuoheti, Linfa Guo, Zhao Chen, Xiaojie Bai, Tongzu Liu

**Affiliations:** ^1^ Department of Urology, Zhongnan Hospital of Wuhan University, Wuhan, China; ^2^ Institute of Hepatobiliary Diseases , Zhongnan Hospital of Wuhan University, Wuhan, China

**Keywords:** urosepsis, rats, animal model, *Escherichia coli*, upper urinary tract obstruction, pathophysiology

## Abstract

Despite extensive research, urosepsis remains a life-threatening, high-mortality disease. Currently, animal models of urosepsis widely accepted by investigators are very scarce. This study aimed to establish a standardized and reproducible model of urosepsis in rats. Forty adult Wistar rats were randomly divided into four groups according to the concentration of injected E. coli suspensions: Sham, Sep 3×, Sep 6×, and Sep 12×. Because the ureter is so thin and fragile, no conventional needle can be inserted into the ureter, which is probably why rats are rarely used to develop models of urosepsis. To solve this problem, the left ureter was ligated in the first procedure. After 24 hours, the left ureter above the ligation was significantly dilated, then saline or different concentrations of E. coli at 3 ml/kg were injected into the left renal pelvis using a 30G needle. The left ureter was subsequently ligated again at a distance of 1 cm from the renal hilum to maintain high pressure in the renal pelvis. Following injection of E. coli or saline for 24 h, three rats from each group were sacrificed and their organs (lung, liver, and right kidney) were collected. In contrast, the remaining seven rats continued to be observed for survival. At 10 days after E. coli injection, rats in the sep12× group had a higher mortality rate (100%) compared to the sep3× group (28.6%) or the sep6× group (71.4%). The significant changes in peripheral blood WBC count, serum IL-6 and TNF-α levels were also in the sep12× group. In addition, rats in the sepsis group showed multi-organ dysfunction, including damage to the lungs, liver, and kidneys. The establishment of a standardized rat model of urosepsis may be of great value for studying the pathophysiological of urosepsis.

## Introduction

Sepsis, a life-threatening organ dysfunction with rapid progression and high mortality (17-26%), is the leading cause of death in critically ill patients worldwide (1–3). Depending on the site of infection, infections originating in the urinary tract and/or male genital tract are referred to as urosepsis (4, 5). It is estimated that approximately 20-30% of all sepsis cases are urosepsis. In total, there are an estimated 31.5 million sepsis cases each year, representing a potential of up to 9.45 million cases of urosepsis (6). Therefore, sepsis and urosepsis have been recognized as very concerning problems by many hospitals and are made a global health priority by the World Health Organization. Despite many new research results in recent years, the pathophysiology of sepsis is still incompletely understood. Several animal models have been created that all seek to mimic the typical pathophysiological changes in septic patients to study the pathophysiological causes of sepsis.

Injection of endotoxin or bacteria, cecum ligation and puncture (CLP), and colonic ascending stent peritonitis (CASP) are the commonly used models of sepsis (7–9). Among these, the rodent cecum ligation and puncture (CLP) model of experimental sepsis has grown to be the most popular and is currently regarded as the gold standard for sepsis research (10–12). However, widely applied and standardized animal models of urosepsis are relatively rare. Some scholars have found that rabbits can be used to develop models of urosepsis by injecting E. coli into the renal pelvis (13–16). Rodents, the most widely used for experimental research, are rarely used to make models of urosepsis. Therefore, the establishment of a standardized rat model of urosepsis may rapidly advance the study of the pathophysiological mechanisms of urosepsis.

In this study, we attempted to utilize rats to produce a standardized and reproducible model of urosepsis by injecting E. coli into the renal pelvis. We evaluated the effectiveness of a rat model of urosepsis by observing survival rates and blood cultures, detecting changes in WBC and inflammatory factors, and verifying multi-organ damage to the lungs, liver, and kidneys. The establishment of a standardized rat model of urosepsis may be of great value for studying the pathophysiological of urosepsis.

## Materials and methods

### Animal

Adult Wistar rats of either sex (weight 250–300 g) were purchased from Beijing Speford Biotechnology Co. (Beijing, China). All experiments were performed at the Animal Research Institute of Zhongnan Hospital at Wuhan University. The animal study was evaluated and approved by the ethics committee of the Zhongnan Hospital at Wuhan University. Rats were raised at controlled temperature (21-25°C) and humidity (45-55%) with a 12-hour light/dark cycle for 7 days to acclimate to the environment.

### Experimental procedures

Forty adult Wistar rats (250-300g) were randomly divided into four groups according to the concentration of injected E. coli suspensions: Sham (injected with saline), Sep 3× (injected with 3×10^8^ cfu/mL E. coli), Sep 6× (injected with 6×10^8^ cfu/mL E. coli), Sep 12× (injected with 12×10^8^ cfu/mL E. coli).

Before the experiment, Wistar rats were fasted overnight but allowed to drink freely. All rats were anesthetized with intraperitoneally injected 30 mg/kg of 1% sodium pentobarbital. After anesthesia, the abdomen of the rats was shaved, and a 3 cm-long incision was performed on the left side of the abdomen. The abdominal cavity of the rats was opened to expose the left kidney, and the left ureter was carefully isolated. At a distance of 2 cm from the left renal hilus, we ligated the left ureter using 4-0 silk, placing the left kidney and intestine back into the abdominal cavity, closing the abdominal cavity, and suturing the skin. 24 h later, the rats were reanesthetized, and the abdominal cavity was reopened, showing that the left renal pelvis and left ureter were significantly dilated compared with those before ligation. Groups Sep3×, 6×, and 12× were injected with 3 ml/kg E. coli solution in the left ureter above the ligation at a concentration of 3×10^8^, 6×10^8^, and 12×10^8^ cfu/ml, respectively. Saline was also injected into the left ureter of the sham group at 3 ml/kg. Subsequently, the left ureter was ligated again at a distance of 1 cm from the renal hilum to maintain a state of pelvic hypertension. The rats were then sutured and received postoperative analgesic meloxicam (1 mg/kg, s.c.).

Blood samples were collected at four postoperative time points (0h, 2h, 24h, and 48h). A portion of the fresh samples was utilized for routine blood testing (WBC count). Additional blood samples were processed at 3000 rpm for 20 minutes using a cryogenic centrifuge, and the supernatant was stored in a -80°C refrigerator for TNF-α and IL-6 assay.

### Bacterial culture

Escherichia coli (E. coli) (ATCC 25922) was purchased from Guangdong Huankai Microbial Technology Co. and cultured on McConkey Agar (Solarbio Life Science Technology Co., Beijing, China) for 24h at 37°C to form individual colonies. Afterward, a single colony of bacteria was picked and inoculated in LB medium at 37°C with shaking at 200 rpm for 18-24h. The bacteria medium was precipitated by centrifugation at 2000g for 10 min and then resuspended in saline to a concentration of 3×10^8^,6×10^8^,12×10^8^ cfu/mL. To ensure accurate concentration, bacterial suspensions were tested using a bacterial turbidimeter (Thermo Fisher Scientific, USA).

After injection of E. coli for 24h, blood samples were collected and inoculated onto MacConkey agar for 24h at 37°C. If pink colonies were found, E. coli in the blood was proven.

### Clinical observations

After intrarenal pelvis injection of E. coli, rats were examined for general responses such as consciousness, activity, weakness, and mortality. Every 4-6 hours, rectal body temperature was measured (Thermometer type T15SGF; Panasonic, Japan), as well as respiratory rate, heart rate, and body weight. Rats were euthanized for humanitarian reasons when they reached a behavior score of 1, where 1 represented Moribund [adapted from Yang et (17)].

### Measurement of WBC, Cytokines(IL-6, TNF-α), Serum CRE, BUN, AST, and ALT

White blood cell (WBC) counts were determined using an automatic hematology analyzer (Nihon Kohden, Japan). Rat serum concentrations of interleukin IL-6 and tumor necrosis factor-alpha (TNF-α) were determined by the Ellisa enzyme immunoassay kit (Wuhan antigene Biotechnology Co., Ltd. Wuhan, China) according to the manufacturer’s protocol. Determination of markers of kidney function by BUN and CRE kits and changes in liver function by AST and ALT kits according to the manufacturer’s instructions (Nanjing Jiancheng Institute of Biotechnology, Nanjing, China)

### Real-time PCR

Under the manufacturer agreement, total RNA was extracted from bladder cancer cells using RaPure Total RNA Micro Kit (Magen, China). The RNA NanoPhotometer spectrophotometer (IMPLEN, Westlake Village, CA, USA) quantified the RNA at 260 nm/280 nm. Following the package recommendations, 2 μg of total RNA was reverse transcribed to cDNA utilizing ABScript II RT Master Mix (ABclonal, Wuhan, China). Using a Bio-Rad (Hercules, CA, USA) CFX96 system, qRT-PCR was used to ascertain the mRNA level of an interesting gene predicated upon SYBR green. The primer sequence is shown in **Table S1**. Each target gene’s relative mRNA expression level was estimated using the 2−ΔΔCT method in conjunction with ACTB as an internal loading control.

### Western blotting analysis

Cells were lysed sufficiently in RIPA that contained 1% protease inhibitor and 1% PMSF (all from Sigma-Aldrich, St. Louis, MO). 40 μg total protein was separated by 10–12.5% SDS-PAGE electrophoresis and transferred onto a polyvinylidene fluoride (PVDF) membrane (Millipore, cat# IPVH00010). After blocking with 5% skim milk at room temperature for 2 h, the membrane was first treated with the primary antibody (**Table S2**) at 4°C for an overnight period, followed by incubation with the secondary antibody—goat anti-rabbit IgG (**Table S3**)—at room temperature for an additional two hours. The bands on the membrane were monitored on a Tanon-5200 ECL imager (Tanon, Shanghai, China) and visualized by an enhanced chemiluminescence kit (Thermo Scientifisher, Waltham, MA, USA).

### Immunohistochemical (IHC)

Formalin-fixed, paraffin-embedded tissue sections were first deparaffinized. And then, endogenous peroxidase activity was inhibited using H2O2. The indicated primary antibody (**Table S2**) and secondary antibody (**Table S3**) were added to the sections according to the suggested methods offered by the manufacturer. All the slides were examined under an inverted microscope at 200× magnification.

### Statistical analysis

All experimental data were represented as the means ± standard error. Student t-tests or one-way ANOVA were employed to assess the statistical analyses, with P < 0.05 regarded as statistically significant.

## Results

### Surgical methods and critical points of the rat urosepsis model

**Figure 1:**
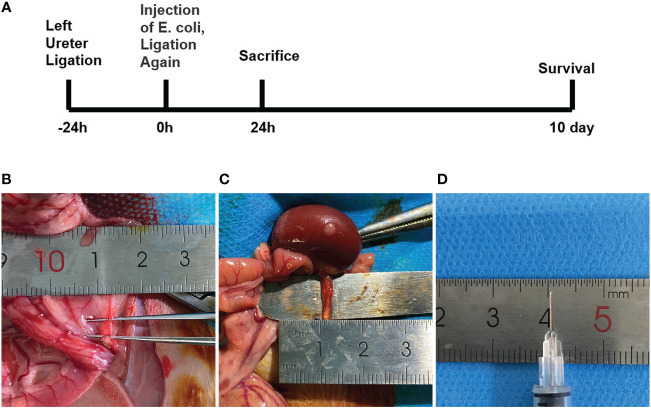
The experimental procedure and critical steps in the process of urinary sepsis model in rats.**(A)** Schematic diagram of the experimental procedure. **(B)** The ureter prior to ligation. **(C)** Dilated ureter after ligation for 24h. **(D)** 30G syringe needle.

To better investigate the mechanism of urosepsis, we tried to establish an animal model of urosepsis in rats. Briefly, we first ligated the left ureter, injected E. coli suspension into the dilated ureter after 24 h, and then ligated the left ureter again at 1 cm from the renal hilum to prevent the flow of E. coli out of the renal pelvis. Three rats in each group were killed at 24 h postoperatively, and the organs were collected, while the remaining seven rats continued to be observed for survival (**Figure 1A**). We found that the ureters of rats were very slim, not even more than 1 mm in diameter (**Figure 1B**), which made it extremely difficult to establish a model of urosepsis. After 24 h of ureteral ligation, the original slender ureter became significantly dilated, and its diameter could reach 3-4 mm(**Figure 1C**). We could easily inject E. coli into the dilated ureter using a syringe with a 30G needle (**Figure 1D**).

### Characterization of urosepsis in rats.

**Figure 2:**
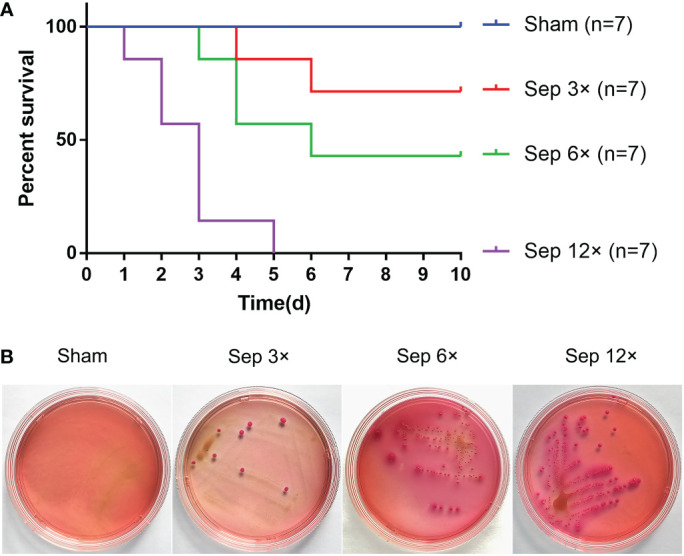
Survival analysis and Blood cultures after ureter ligation and inoculation of E coli. **(A)** Survival analysis after ureter ligation and inoculation of *E coli*. **(B)** Blood specimens were incubated on MacConkey agar at 37°C after 24h inoculation with *E coli*.

**Figure 3:**
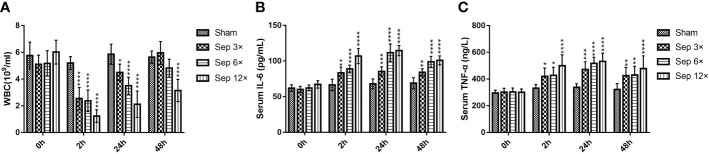
Changes in WBC **(A)**, serum IL-6 **(B)**, and serum TNF-α **(C)** in rats at different E coli concentrations and intervals. Values were shown as mean ± SD. *P < 0.05, **P < 0.01, ***P <0.001, and ****P < 0.0001 vs sham group.

We detected that greater concentrations of E. coli led to higher mortality in rats at 10 days, with 28.6% mortality in the sep 3× group, 71.4% mortality in the sep 6× group, and 100% mortality in the sep 12× group (**Figure 2A**). After 24 h of E. coli or saline injection, all rats were tested by blood culture. **Figure 2B** showed that blood cultures were positive in the three sepsis groups while negative in the sham group. In addition, we collected blood samples at four-time points (0h, 2h, 24h, and 48h postoperatively) for WBC counts. And the data performed statistically significant changes in WBC of all rats 2h after renal pelvis injection of E. coli solution compared to preoperative values except for the control group. These changes were most evident in the sep12× group with a mean ± SD WBC of 1.24 ± 0.47× 109/L (**Figure 3A**). Similarly, the changes in IL-6 and TNF-α levels were most remarkable in the sep12× group with mean± SD values of 114.78 ± 7.18 pg/mL and 531.46± 61.99 ng/L, respectively. It is important to note that the increase in IL6 and TNF-α concentrations occurred at 24h postoperatively instead of 2h (**Figures 3B, C**).

### Lung injury in the rat model of urosepsis

**Figure 4:**
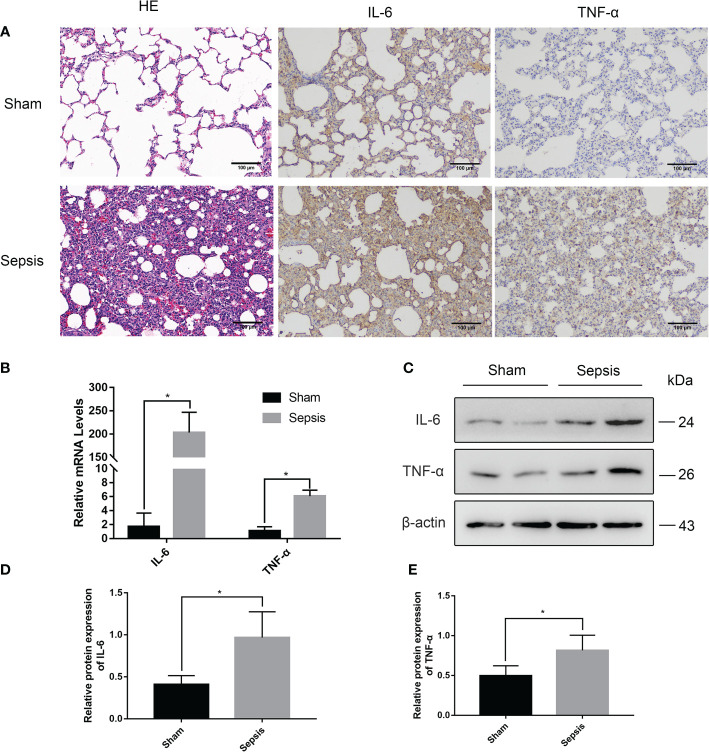
Lung injury in the rat model of urinary sepsis. **(A)** Representative images of hematoxylin and eosin **(H&E)** staining of lung tissue from the sham and sepsis groups, immunohistochemical analysis of IL-6 and TNF-α in lung tissue. **(B)** The relative mRNA levels of IL-6 and TNF-α in the lung tissue. **(C)** Expressions of IL-6 and TNF-α in lung tissue were tested by western blot, and β-actin was used as a loading control. **(D, E)** Quantitative data of the levels of IL-6 and TNF-α. The sep 6× group was selected as a representative of the sepsis groups. Values were shown as mean ± SD. *P < 0.05, vs sham group.

The sep 6× group was selected as a representative of the sepsis groups compared with the sham group. HE staining examination (**Figure 4A**) showed that the alveolar wall was widened, and the alveolar lumen collapsed in most areas due to edema in the sepsis group. At the same time, the alveolar wall and alveolar lumen of the sepsis group also had a large number of inflammatory cell infiltrates and erythrocyte exudates. Immunohistochemical (IHC) analysis of paraffin-embedded lungs illustrated that the levels of IL-6 and TNF-α were considerably greater in the sepsis group than in the sham group (**Figure 4A**). As shown in **Figure 4B**, there was a statistically significant difference in the relative mRNA levels of IL-6 and TNF-α in the lung tissue of the sepsis group vs the sham group (P<0.05). Furthermore, compared with the sham group, Western blotting analysis of the collected lung tissues revealed that the expression levels of IL-6 and TNF-α proteins were increased in the model sepsis group (**Figures 4C–E**).

### Liver injury in the rat model of urosepsis

**Figure 5:**
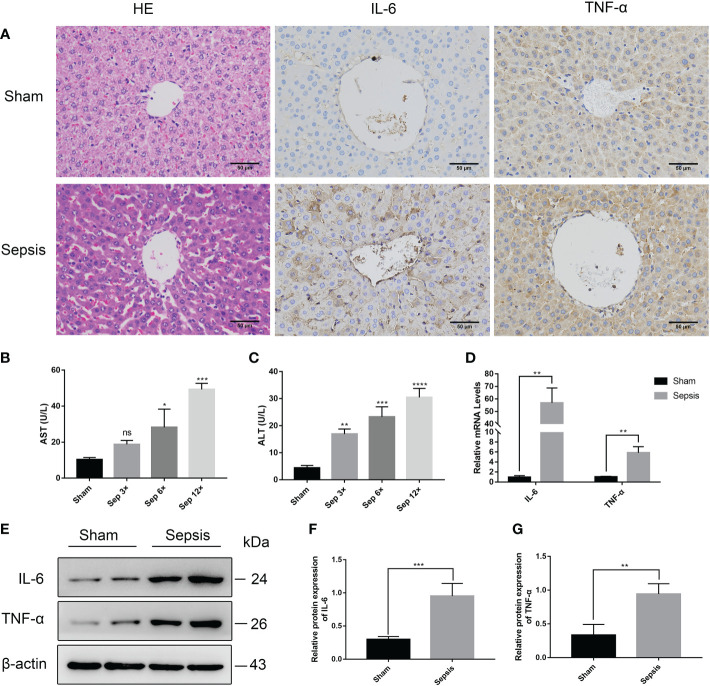
Liver injury in the rat model of urinary sepsis **(A)** Representative images of hematoxylin and eosin **(H&E)** staining of liver tissue from the sham and sepsis groups, immunohistochemical analysis of IL-6 and TNF-α in liver tissue (magnification, 200×; bar). **(B, C)** Serum AST and ALT levels in rats 24 hours after E. coli or saline inoculation of the renal pelvis. **(D)** The relative mRNA levels of IL-6 and TNF-α in the lung tissue. **(E)** Expressions of IL-6 and TNF-α in liver tissue were tested by western blot, and β-actin was used as a loading control. **(F, G)** Quantitative data of the levels of IL-6 and TNF-α. The sep 6× group was selected as a representative of the sepsis groups. Values were shown as mean ± SD. *P < 0.05, **P < 0.01, ***P <0.001, and ****P < 0.0001 vs sham group. "ns" means "not signifcant".

Histopathological examination proved that liver sections from sepsis rats had features of liver injury, such as inflammatory infiltration, disorganized cell arrangement, vacuolated necrosis, and ductal hyperplasia(**Figure 5A**). And serum levels of ALT and AST were elevated (compared to sham surgery) due to sepsis-induced liver injury (**Figures 5B, C**). In addition, we also performed a reverse transcription-polymerase chain reaction (RT-PCR) analysis on the livers of rats (**Figure 5D**). The relative mRNA levels of IL-6 and TNF-α were significantly higher than those of the sham group (P<0.01). Immunohistochemistry and Western blotting analysis of liver tissue also revealed that the levels of IL-6 and TNF-α protein expression were considerably greater in the sepsis group (**Figures 5A–G**).

### Kidney injury in the rat model of urosepsis

**Figure 6:**
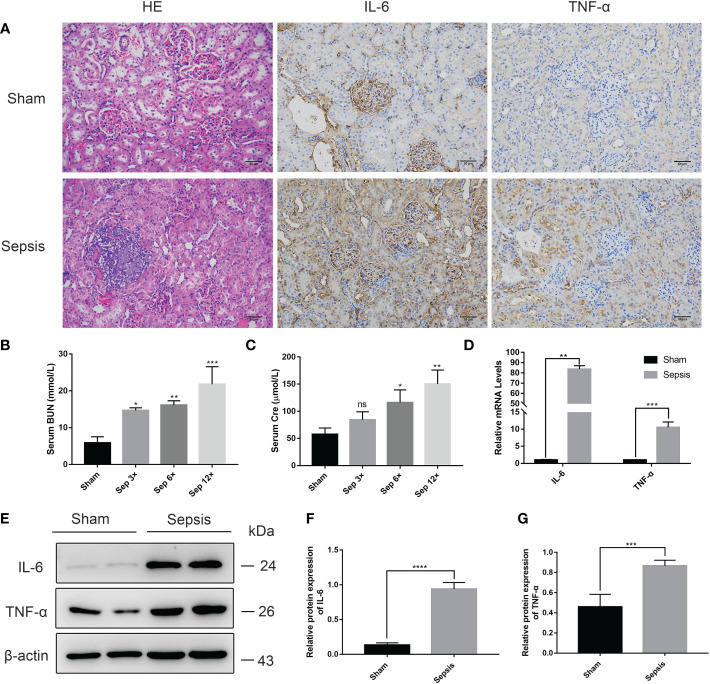
Kidney injury in the rat model of urinary sepsis **(A)** Representative images of hematoxylin and eosin **(H&E)** staining of kidney tissue from the sham and sepsis groups, immunohistochemical analysis of IL-6 and TNF-α in kidney tissue (magnification, 200×; bar, 50 μm). **(B, C)** Serum BUN and Cre levels in rats 24 hours after E. coli or saline inoculation of the renal pelvis. **(D)** The relative mRNA levels of IL-6 and TNF-α in kidney tissue. **(E)** Expressions of IL-6 and TNF-α in kidney tissue were tested by western blot, and β-actin was used as a loading control. **(F, G)** Quantitative data of the levels of IL-6 and TNF-α. The sep 6× group was selected as a representative of the sepsis groups. Values were shown as mean ± SD. *P < 0.05, **P < 0.01, ***P <0.001, and ****P < 0.0001 vs sham group. "ns" means "not signifcant".

We performed HE staining of the right kidney tissue and discovered that renal tissue sections from the sepsis group had apparent features of renal injury, such as vacuolar degeneration of renal tubular epithelial cells, separation of renal tubular epithelial cells, and inflammatory cell infiltration (**Figure 6A**). Using measurements of serum biochemical parameters in rats, we demonstrated that serum Cre and BUN were significantly increased in the sepsis group 24 h after E. coli injection (**Figures 6B, C**). The most significant elevation of serum Cr and BUN was examined in the sep 12× group compared to the sham group. In addition, RT-PCR analysis of rat livers (**Figure 6D**) revealed that the relative mRNA levels of IL-6 and TNF-α were considerably greater than those in the sham group. Finally, we identified that IL-6 and TNF-α protein expression levels were significantly elevated in the sepsis group by immunohistochemistry and Western blotting analysis of renal tissues compared to the sham group (**Figures 6A, 6E–G**).

## Discussion

This research depicts a standardized rat model of urosepsis with ligation of one ureter and consequent injection of Escherichia coli. The experimental results demonstrate our success in developing a standardized model of urosepsis in rats and confirm a significant correlation between the severity of urosepsis and the concentration of inoculated E. coli.

The incidence of urosepsis is increasing approximately 8.7% per year, which is closely linked to the widespread availability of upper urinary tract endoscopic procedures (18). In addition to stone co-infection and prolonged surgery, high intraoperative pelvic pressure leading to bacterial entry into the bloodstream is also a factor contributing to the development of urosepsis (19). Nguyen et al. (20) demonstrated that when the renal pelvic pressure exceeds 40 cm H_2_O, the urine can reflux into the bloodstream carrying bacteria and metabolic waste products. Wu et al. (13) discovered that E. coli was injected into the renal pelvis at 2 ml/kg to maintain intrapelvic hypertension, leading to the development of a New Zealand rabbit model of urosepsis. In a preliminary pre-experiment, we observed low mortality in rats when 2 ml/kg of E. coli was injected into the renal pelvis. We hypothesized that intrapelvic injection of a small number of bacteria leads to a pyelonephritis model rather than a urosepsis model because of insufficient pressure in the renal pelvis. Gupta K et al. established pyelonephritis models by injecting small amounts of bacteria into the renal pelvis of rats or rabbits (21, 22), which confirmed our hypothesis. After several attempts, we finally chose to inject 3ml/kg of E. coli into the renal pelvis. Autopsy of the dead rats revealed that the left kidney was enlarged while renal parenchyma was thinned, and the renal capsule was intact without rupture, indicating that the injection dose of 3 ml/kg was safe. Therefore, we believe that maintaining renal pelvic hypertension is necessary for this urosepsis model.

CLP is similar to the clinical development of human sepsis, which is why it has been called the gold standard for sepsis research (23). The model features a pathogen from the host interior and mimics the pathogenesis of peritonitis (24). However, the severity of sepsis is not well standardized due to the difficulty of quantifying surgical operations, such as the percentage of cecum ligation and the dose of bacteria that enter the peritoneum after the puncture (25). Therefore, Rittirsch et al. renormalized the details of the CLP technique to control the severity of sepsis by the length of the ligated cecum and the size and/or the number of punctures (26). In our study, we obtained a similar mortality rate using ligation of the ureter 1 cm from the renal hilum to standardize the surgical procedure. Compared to the CLP technique of regulating the ligation site of the cecum, it is undoubtedly much simpler to adjust different concentrations of E. coli to achieve control of sepsis severity in our experiments.

The host inflammatory response might be seen as a balanced response between pro-inflammatory and anti-inflammatory mediators (27). In the early stages of sepsis, activation of the host’s innate immune system leads to a massive release of pro-inflammatory mediators, the main ones including IL-6 and TNF-α, as well as chemokines (24, 28). In various animal models of sepsis, IL-6 and TNF-α expression were significantly elevated compared to the normal group (29–31). Besides, plasma IL-6 levels have been found to be potential indicators of the intensity of inflammation and mortality predictions (32). As the concentration of inoculated E. coli increased, we observed a significant increase in serum IL-6 and TNF-α levels, indicating that adjusting the concentration of E. coli could control the severity of sepsis in our study.

In contrast, peripheral blood WBC counts were significantly decreased in the Sep 12× group at 2 h postoperatively after E. coli inoculation. Wu et al. (13) revealed a decrease in WBC counts 2 h after formation in a New Zealand rabbit model of urosepsis, which is consistent with our results.

In 2016, sepsis was redefined as life-threatening organ dysfunction resulting from dysregulated host responses to infection (33). The severity of organ dysfunction is quantified using the Sequential Organ Failure Assessment (SOFA) score (34). Compared to the previous definition, the new version of the definition of sepsis places more emphasis on multi-organ damage. Our data suggested that the relative mRNA levels and protein levels of inflammatory factors (IL-6 and TNF-a) extracted from three organs—the lung, liver, and kidney—were considerably greater in the sepsis group than in the sham group. Our rat urosepsis model confirmed three organ damages by HE staining, immunohistochemistry, RT-PCR, and Western blotting.

## Conclusions

Current animal sepsis models do not fully replicate the pathophysiological process of human sepsis. In this study, our novel rat sepsis model simulates human urosepsis due to upper urinary tract obstruction combined with urinary tract infection. In addition, this model can manipulate the severity of sepsis by adjusting the concentration of E. coli suspensions. Therefore, this rat model may be an essential tool for studying the pathophysiological mechanisms of sepsis or urosepsis.

## Data availability statement

The original contributions presented in the study are included in the article/**Supplementary Material**. Further inquiries can be directed to the corresponding authors.

## Ethics statement

The animal study was reviewed and approved by Zhongnan Hospital of Wuhan University’s Ethics Committee.

## Author contributions

YC, CB, and PS designed the study, analyzed the data, and prepared the original draft. YC, XY, and PZ collected the data and conducted the statistical analysis. ZY, PL, and KT collected the data. LG and ZC raised rats. YC wrote the paper. XB and TL designed and monitored the study, together with significant revisions to the manuscript. All authors have approved the final draft submitted.
