# Supplementary material for: A novel model of urosepsis in rats developed by injection of Escherichia coli into the renal pelvis
Source: Front Immunol. 2023 Jan 5;13:1074488. doi: 10.3389/fimmu.2022.1074488 (PMC9849364; doi:10.3389/fimmu.2022.1074488)
Supplement: Supplementary file 1 [file Table_1.docx]

**Supplementary Material**

**Supplementary Tables**

Supplementary table S1 Primer sequences for qRT-PCR

| Gene (Human) | Forward primer (5’~3’) | Reverse primer (5’~3’) |
| --- | --- | --- |
| ACTB | AGATCAAGATCATTGCTCCTCCT | ACGCAGCTCAGTAACAGTCC |
| IL-6 | CACTTCACAAGTCGGAGGCT | AGCACACTAGGTTTGCCGAG |
| TNF-α | GGCTTTCGGAACTCACTGGA | CCCGTAGGGCGATTACAGTC |

Supplementary table S2 Primary antibodies for Western Blot and Immunohistochemical

| Antigens (Human) | Species antibodies raised in | Dilution | Supplier |
| --- | --- | --- | --- |
| IL-6 | Rabbit, polyclonal | 1:1000 (WB)  1:200 (IHC) | Affinity #DF6087 |
| TNF-α | Rabbit, polyclonal | 1:1000 (WB)  1:200 (IHC) | Affinity #AF7014 |
| β-actin | Rabbit, monoclonal | 1:1000 (WB) | Abclonal AC038 |

Supplementary table S3 Secondary antibodies for Western Blot and Immunohistochemical

| Secondary detection system used | Species antibodies raised in | Dilution | Supplier |
| --- | --- | --- | --- |
| Anti-Rabbit-IgG (H+L)-HRP | Goat | 1:10000 (WB) | Abclonal, USA, Cat. AS014 |
| Anti-rabbit IgG H&L(HRP) | Goat | 1:2000 (IHC) | Abcam, USA, cat. ab205718 |
